# Comparison of a nurse initiated insulin infusion protocol for intensive insulin therapy between adult surgical trauma, medical and coronary care intensive care patients

**DOI:** 10.1186/1471-227X-7-14

**Published:** 2007-08-29

**Authors:** Melissa M Barth, Lance J Oyen, Karen T Warfield, Jennifer L Elmer, Laura K Evenson, Ann N Tescher, Philip J Kuper, Michael P Bannon, Ognjen Gajic, J Christopher Farmer

**Affiliations:** 1 Department of Nursing, Mayo Clinic, Rochester, Minnesota, USA; 2 Pharmacy Services, Mayo Clinic, Rochester, Minnesota, USA; 3 Department of Surgery, Mayo Clinic, Rochester, Minnesota, USA; 4 Department of Medicine, Mayo Clinic, Rochester, Minnesota, USA

## Abstract

**Background:**

Sustained hyperglycemia is a known risk factor for adverse outcomes in critically ill patients. The specific aim was to determine if a nurse initiated insulin infusion protocol (IIP) was effective in maintaining blood glucose values (BG) within a target goal of 100–150 mg/dL across different intensive care units (ICUs) and to describe glycemic control during the 48 hours after protocol discontinuation.

**Methods:**

A descriptive, retrospective review of 366 patients having 28,192 blood glucose values in three intensive care units, Surgical Trauma Intensive Care Unit (STICU), Medical (MICU) and Coronary Care Unit (CCU) in a quaternary care hospital was conducted. Patients were > 15 years of age, admitted to STICU (n = 162), MICU (n = 110) or CCU (n = 94) over 8 months; October 2003-June 2004 and who had an initial blood glucose level > 150 mg/dL. We summarized the effectiveness and safety of a nurse initiated IIP, and compared these endpoints among STICU, MICU and CCU patients.

**Results:**

The median blood glucose values (mg/dL) at initiation of insulin infusion protocol were lower in STICU (188; IQR, 162–217) than in MICU, (201; IQR, 170–268) and CCU (227; IQR, 178–313); *p *< 0.0001. Mean time to achieving a target glucose level (100–150 mg/dL) was similar between the three units: 4.6 hours in STICU, 4.7 hours in MICU and 4.9 hours in CCU (*p *= 0.27). Hypoglycemia (BG < 60 mg/dL) occurred in 7% of STICU, 5% of MICU, and 5% of CCU patients (*p *= 0.85). Protocol violations were uncommon in all three ICUs. Mean blood glucose 48 hours following IIP discontinuation was significantly different for each population: 142 mg/dL in STICU, 167 mg/dL in MICU, and 160 mg/dL in CCU (*p *< 0.0001).

**Conclusion:**

The safety and effectiveness of nurse initiated IIP was similar across different ICUs in our hospital. Marked variability in glucose control after the protocol discontinuation suggests the need for further research regarding glucose control in patients transitioning out of the ICU.

## Background

Sustained hyperglycemia is a known risk factor for adverse outcomes in the critically ill patient, whether or not the patient has a history of diabetes mellitus [1-3]. There are many factors that affect glycemic control; metabolic derangement and counter-regulation, increased stress, decrease insulin (resistance or underproduction), increased glucose administration just to name a few [4,5]. Although short term glycemic increases related to stress are not associated with rise in mortality in all populations [1,6,7].

Intensive insulin therapy is emerging as a treatment modality. At the time of our study, other than cardiovascular surgery patients [1,8,9], it was uncertain what specific patient populations benefit from intensive insulin therapy. Other populations are beginning to emerge in the literature [7]; trauma patients [10,11], intraoperative [12], and medical patients [13,14].

Despite some uncertainties, the management of hyperglycemia utilizing insulin protocols is fast becoming a new standard in critical care practice [1,13-23]. Nurse initiated protocols generally have been found to improve patient care [13,17,22], limit the prescribing variability [15,16,18,20,24] and contribute to financial cost savings [17]. However it is unknown if a nurse initiated IIP is effective and safe across different ICU populations. Also, it appears similar protocols in different populations' yields different clinical outcomes [9,14]. It is unclear if the protocol or operation of the protocol leads in part to these differences.

In this study we sought to determine if the IIP was effective and safe in bringing blood glucose values within the target range of 100–150 mg/dL among the three different ICUs (surgical, medical and coronary care). We were also interested in glycemic control across different ICU populations in the first 48 hours after the IIP was discontinued, when the patients transition out of the ICU.

## Methods

### Design

We conducted a descriptive retrospective review of 366 patients having 28,192 blood glucose values in the analysis of outcomes after Mayo Foundation Institutional Review Board (IRB) approval was granted. Patients had been cared for in one of three ICUs in a quaternary care hospital at Mayo Clinic Rochester, MN where an intensive insulin protocol was utilized. The ICUs included a 24-bed Surgical Trauma Intensive Care Unit (STICU), a 24-bed Medical ICU (MICU), and a 16-bed Coronary Care Unit (CCU).

### Patients

Inclusion criteria included adult patients, 15 years of age and older, admitted to STICU, MICU or CCU who had a blood glucose level greater than 150 mg/dL while on the unit and were placed on an IIP during the identified data collection time frame October 2003-June 2004. 366 out of 386 eligible patients were used for data analysis. Eighteen patients were excluded since they did not grant approval for use of their data for research purposes. One patient was not started on insulin drip and one was on a modified insulin drip prior to ICU admission. There were not any other exclusion criteria. Patients readmitted to the ICU during the same or subsequent hospitalization were not considered for analysis. Patient characteristics are summarized in Table 1.

**Table 1:** Patient characteristics

	**STICU (n = 162)**	**MICU (n = 110)**	**CCU (n = 94)**	***p *Value**
Age, mean (SD)	59.9 (19.5)	61.4 (17.6)	67.8 (12.7)	0.01
Gender, Female, n (%)	79 (49)	57 (52)	35 (37)	0.09
Weight, kg, mean (SD)	90.5 (32.9)	84.7 (25.8)	87.4 (23.0)	0.70
Height, cm, mean (SD)	169.0 (9.6)	167.4 (19.4)	170.5 (10.2)	0.44
Diabetes, n (%)	48 (30)	47 (43)	57 (61)	< 0.0001
Medications				
IV/Oral Corticosteroids, n (%)	34 (21)	51 (46)	9 (10)	< 0.0001
Epinephrine/Norepinephrine, n (%)	13 (8)	16 (15)	14 (15)	0.14
Beta blockers, n (%)	57 (35)	25 (23)	52 (55)	< 0.0001
APACHE III score, mean (SD)	n = 158 52.7 (21.9)	n = 109 67.3 (30.1)	n/a	< 0.0001
ICU LOS, days, median (IQR)	6.4 (3.3–13.2)	2.9 (1.3–8.7)	2.4 (1.3–4.2)	< 0.001
ICU mortality, n (%)	8 (5)	16 (15)	15 (16)	0.004
Hospital mortality, n (%)	12 (7)	21 (19)	25 (27)	< 0.001

ICU, Intensive Care Unit; STICU, Surgical Trauma ICU; MICU, Medical ICU; CCU, Coronary Care Unit; APACHE, Acute Physiological and Chronic Health Evaluation; n/a, not available; LOS, length of stay; IQR, interquartile range

### Interventions

#### Intensive Insulin Infusion Protocol for Insulin Therapy

A 4-column, non-calculated protocol for continuous intravenous insulin administration (measured in units/hour) on adult patients was initiated by the RN with blood glucose value greater than 150 mg/dL [see Additional file 1].

#### Glucose Meter for Blood Sampling

Blood glucose values collected by vascular access team (VAT) laboratory personnel, obtained by capillary "fingerstick", arterial or venous blood draw (measured in mg/dL) were measured via one of three methods: 1) Bedside glucose values were measured using a standard hospital glucose meter (SureStep Flexx; Lifescan, Johnson & Johnson, New Brunswick, NJ). 2) Serum glucose values were measured using a standard hospital glucose instrument (Modular Roche Hitachi, Japan, Roche Corporation, Indianapolis, IN). 3) Plasma glucose values were measured using a standard hospital glucose instrument (Beckman Glucose Analyzer 2, Beckman Coulter, Brea, CA). It is estimated that 80% of blood glucose values were obtained by capillary, 19% by arterial and 1% by venous blood draw.

### Definitions

*Time to achieve goal: *hours until first blood glucose value ≤ 150 mg/dL

*Safety: *percent of blood glucose values ≥ 60 mg/dL

*Hyperglycemic: *blood glucose > 150 mg/dL

*Hypoglycemic: *blood glucose < 60 mg/dL

*Mean Percent: *Reflects the average per-patient percent of blood glucose values meeting a specified criterion (e.g. values > 150 or < 60 mg/dL, falling outside the target IIP range), computed from the data from all individuals in the group of interest. To compute this value, the total number of blood glucose values meeting the criterion for a given patient were summed up, divided by the total number of blood glucose measurements, and multiplied by 100. The mean percent was then computed as the simple average of the per-patient percentages for all subjects in the group of interest. This measure was used to reflect the total proportion of the time that study participants had blood glucose measurements outside the targeted IIP range.

### Procedure

At our institution, following the publication of the Van den Berghe study [1], a group of nurses and pharmacists began exploring a project to examine if use of the IIP would make a difference in patient populations other than cardiovascular surgery. A recent pilot study was done on two cardiovascular surgery ICUs utilizing the RN initiated IIP which they adapted from a published protocol [15]. STICU, MICU and CCU ICU populations were utilized to determine if IIP would work similarly in different patient populations. Input and feedback were gathered related to management of patients on insulin. Collaboration with RNs, physicians & pharmacists identified need for better control of critically ill patient blood glucose levels and support was obtained from physician leadership of each ICU. Since the IIP was a new protocol being utilized in STICU, MICU and CCU, staff registered nurses (RNs) were educated prior to implementation. Data collection began one month after education.

A target goal range of 100–150 mg/dL was established for our institutions IIP. Caution to avoid hypoglycemia was one factor that contributed to this range. This factor has been documented in the literature for other institutions as well. Total elimination of hypoglycemia is a desirable but difficult to achieve expectation of critically ill patients due to the confounding factors that contribute to their illness. Our institution has a hypoglycemic protocol to treat patients whose blood glucose falls below 60 mg/dL.

Frequency of blood glucose measurements was determined by the IIP. Hourly values were obtained until the patient was in goal range for 4 continuous hours, and then the frequency was decreased to every two hours. Return to hourly monitoring occurred with the following identified situations: change in nutritional or clinical status, blood glucose above goal range for 2 consecutive readings or blood glucose below 100 mg/dL.

Additional investigator developed tools utilized for data collection included an *IIP Data Collection Tool *(completed by the staff RN) and an *IIP Clinical Nurse Specialist (CNS)/Nursing Education Specialist (NES) Data Collection Tool *which was completed after the protocol was discontinued. The IIP was discontinued at the time the patient transferred out to a general care floor, when ICU care was no longer deemed necessary. One investigator was present on each unit at least 5 days of the week to answer questions & clarify use of protocol during the data collection period. Generally, in the 48 hours after protocol discontinuation, blood glucose monitoring continued at a lower frequency (2–4 times daily) based on glucose values, clinical situations or symptoms. There was variability in treatment ranging from higher goal insulin infusion (not IIP), subcutaneous insulin regimens, oral diabetic agents, or no treatment. Patient follow-up was determined by the primary physician service.

### Statistical analysis

**Figure 1:**
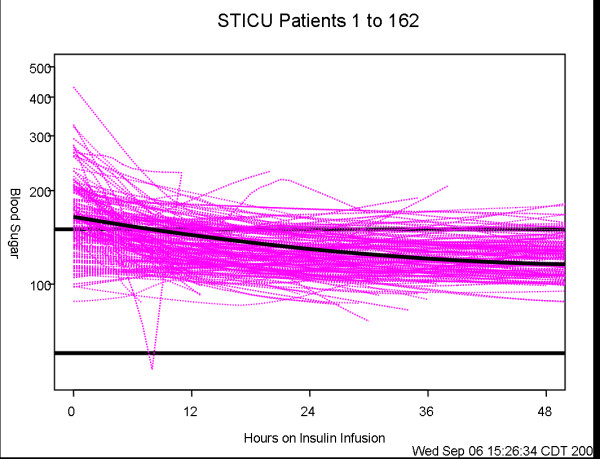
**Individual profiles of blood glucose of patients who underwent insulin infusion-STICU**. Individual profiles of blood glucose of patients who underwent insulin infusion after admission to the STICU, n = 162 (fuchsia lines) along with the predicted level of blood glucose (solid line). The predicted level of blood glucose is based on the mathematical model below where time is measured in days since the start of insulin infusion: Blood glucose (mg/dL) = exp(5.1030 - 0.3023*time + 0.0640*time*time).

**Figure 2:**
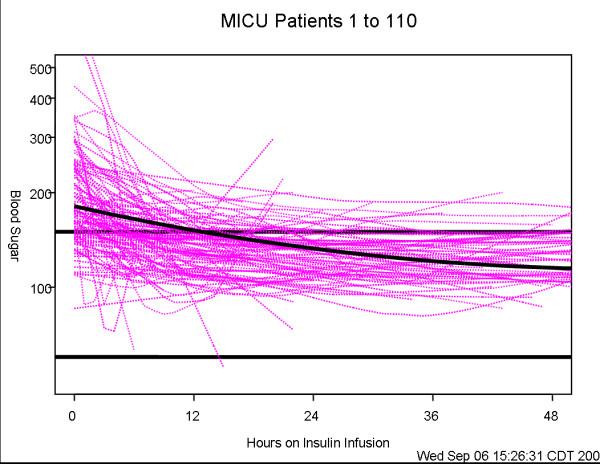
**Individual profiles of blood glucose of patients who underwent insulin infusion-MICU**. Individual profiles of blood glucose of patients who underwent insulin infusion after admission to the MICU, n = 110 (fuchsia lines) along with the predicted level of blood glucose (solid line). The predicted level of blood glucose is based on the mathematical model below where time is measured in days since the start of insulin infusion: Blood glucose (mg/dL) = exp(5.1977 - 0.3936*time + 0.0840*time*time).

**Figure 3:**
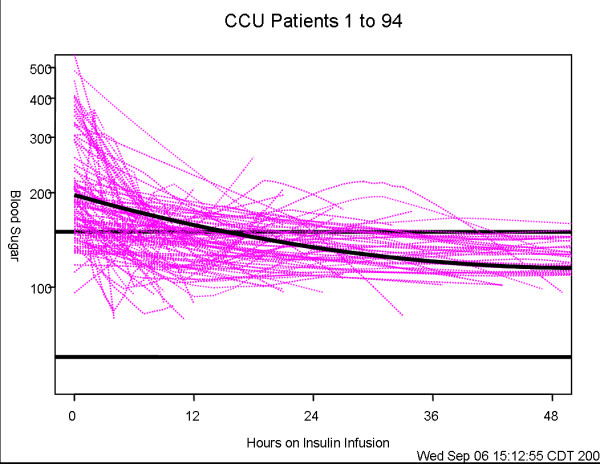
**Individual profiles of blood glucose of patients who underwent insulin infusion-CCU**. Individual profiles of blood glucose of patients who underwent insulin infusion after admission to the CCU, n = 94 (fuchsia lines) along with the predicted level of blood glucose (solid line). The predicted level of blood glucose is based on the mathematical model below where time is measured in days since the start of insulin infusion: Blood glucose (mg/dL) = exp(5.2808 - 0.4987*time + 0.1160*time*time).

**Figure 4:**
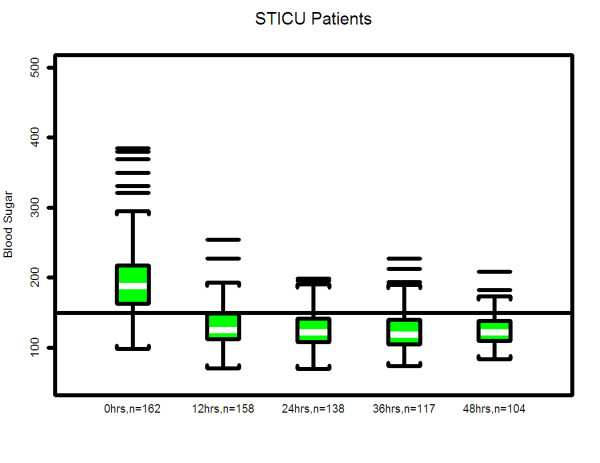
**Median & interquartile range of actual blood glucose values of patients who underwent insulin infusion after admission-STICU**. The box plots indicate the distribution of the individual patient means. See statistical methods section for details.

**Figure 5:**
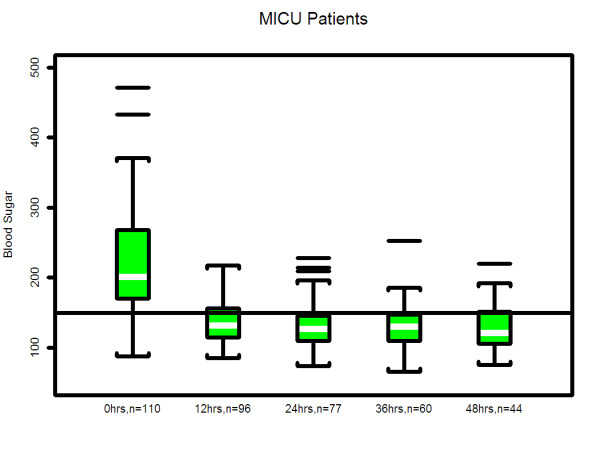
**Median & interquartile range of actual blood glucose values of patients who underwent insulin infusion after admission-MICU**. The box plots indicate the distribution of the individual patient means. See statistical methods section for details.

**Figure 6:**
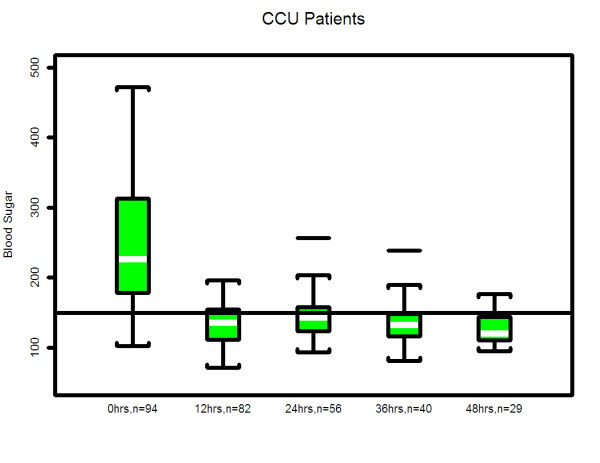
**Median & interquartile range of actual blood glucose values of patients who underwent insulin infusion after admission-CCU**. The box plots indicate the distribution of the individual patient means. See statistical methods section for details.

Summary patient features, reported in Table 1, were calculated for each of the three care units using means (standard deviations), or median (interquartile range) or count (percentage) as appropriate for the data. These variables were compared among the three units using chi-square tests for categorical data, and Kruskal-Wallis tests for continuous data. Comparisons of blood glucose decline among the units are shown by the profile plots in Figures 1, 2, 3. These figures were generated using a mixed model analysis of variance. Specifically, the blood glucose level was modeled as a quadratic function of time (days) on insulin infusion taking into account the type of ICU and the serial aspect of the data. All blood glucose measurements from 2 hours prior to drip start until the order discontinuation time was used for data modeling. To decrease the influence of patients with prolonged ICU stay, any patient who underwent insulin infusion for more than 100 hours had their last measure truncated at 100 hours. As within patient blood glucose measurements could decrease over a wide range, say from 400 mg to 100 mg; a natural logarithm transformation to stabilize the variability of blood glucose was employed. The fitted data was examined for a quadratic association with time by inspecting the graphs which indicated that a quadratic association with time provided a reasonable fit to the data. The final model includes linear and quadratic terms for time, and an indicator variable for ICU type and the interactions between time and ICU type. Thus, the resulting models are multiplicative and curvilinear, meaning that a day on infusion results in a percent decrease in blood glucose but the decrease changes over days. Measurements of blood glucose which were made repeatedly over time were summarized by calculating the patients' mean blood glucose, during a specific interval, and calculating a series of box plots for each unit (Figures 4, 5, 6). The box plots indicate the distribution of the individual patient means. In addition to examining data collected during the IIP, measures of blood glucose obtained in the 48 hours following IIP were obtained, summarized and compared among units in the same manner as described for the data collected during the use of the IIP. The SAS statistical software (SAS Institute Inc, Cary NC) was used for all analyses, and two-sided *p *values < 0.05 were considered to be statistically significant.

## Results

Three hundred and sixty-six patients met inclusion criteria over the eight months of the study period, 162 in STICU, 110 in MICU and 94 in the CCU. General patient characteristics are listed in Table 1. The average length of ICU stay, and the duration of IIP infusion varied, with STICU being the longest. The number of blood glucose measurements obtained while on the IIP compared to the 48 hours after IIP discontinuation were 13,120 (average 81 per patient)/1,926 in STICU, 6,639 (60 per patient)/1,322 in MICU, and 4,199/986 (45 per patient) in CCU. The total blood glucose values utilized for analysis was 28,192. The median blood glucose values at initiation of IIP were statistically different for each unit, STICU, MICU, and CCU (Table 2). Mean insulin infusion rates, however, were similar in all 3 units. While we observed small differences in the time-response curves and individual blood glucose readings at the 24 hour time point, the overall safety and efficacy of the protocol were similar in all three ICUs (Table 2, Figures 1, 2, 3, 4, 5, 6). Deviations from the protocol were uncommon and similar across the 3 ICUs (Table 3).

**Table 2:** Summary data while on the insulin infusion protocol

	**STICU (n = 162)**	**MICU (n = 110)**	**CCU (n = 94)**	***p *Value**
Blood glucose at initiation of IIP, median (IQR), mg/dL	188 (162–217)	201 (170–268)	227 (178–313)	< .0001
Insulin infusion rates, mean (SE)*, ml/hr	1.7 (0.10)	1.7 (0.12)	2.0 (0.13)	0.19
Hours until BG ≤ 150 mg/dL, mean (SD)	n = 159 4.6 (4.4)	n = 103 4.7 (4.4)	n = 89 4.9 (3.9)	0.27
Patients > 150 mg/dL @ time point, n (%):				
12 ± 2 hours	37/158 (23)	28/96 (29)	24/82 (29)	0.49
24 ± 2 hours	18/138 (13)	13/77 (17)	20/56 (36)	0.001
36 ± 2 hours	19/117 (16)	12/60 (20)	9/40 (23)	0.63
48 ± 2 hours	15/104 (14)	12/44 (27)	6/29 (21)	0.18
Duration IIP use, days, mean (SD)	3.8 (3.8)	2.8 (2.7)	2.0 (2.5)	< 0.0001
Patients with at least one blood glucose < 60 mg/dL, n (%)	11 (7)	6 (5)	5 (5)	0.85
Mean percent BG < 60 mg/dL, mean (SD)	0.2 (1.4)	0.3 (1.5)	0.1 (0.6)	0.88

IQR, interquartile range; BG, blood glucose; SE, standard error

*To account for multiple values measured per person

**Table 3:** Deviations from insulin infusion protocol

	**STICU (n = 162)**	**MICU (n = 110)**	**CCU (n = 94)**
Started in a wrong column	27	15	15
Jumped a column	0	0	1
BG < 150 mg/dL when IIP started	4	2	1
Patients with deviation, n (%)	31 (19)	17 (15)	17 (18)

BG, blood glucose

A large percentage of patients in each of the ICUs experienced hyperglycemia after the IIP was discontinued and the patient transitioned out of the ICU. Table 4 summarizes the results 48 hours after the IIP discontinuation.

**Table 4:** Forty-eight hours after insulin infusion protocol discontinued or patient transfer

	**STICU (n = 149)**	**MICU (n = 99)**	**CCU (n = 83)**	***p *Value**
Mean percent of BG > 150 mg/dL, mean (SD)	26 (28)	43 (32)	38 (29)	< 0.0001
BG measurements, mean (SD)	n = 1926	n = 1322	n = 986	
	17.5 (17.0)	15.2 (13.9)	13.5 (10.7)	0.02
BG, mean (SE)*, mg/dL	142 (3.6)	167 (4.4)	160 (4.8)	< 0.0001

BG, blood glucose; SE, standard error

*To account for multiple values measured per person

## Discussion

To our knowledge, this is one of the first studies to compare use of a non-calculating insulin infusion protocol in three different adult ICU patient populations. At the initiation of the protocol, hyperglycemia was more severe in medical than in surgical ICU patients. The protocol effectiveness and safety were, however, similar across the three ICUs. A large percentage of patients developed hyperglycemia after IIP discontinuation.

In our study, hyperglycemia in medical patients may be attributed to a higher incidence of diabetes in CCU & MICU as compared to STICU. In addition, there was higher use of IV or oral corticosteroids in MICU and beta blocker use in CCU. However, despite different starting blood glucose values, the finding of mean time (hours) that the patients blood glucose value first dropped to < 150 mg/dL was similar in all three units (STICU 4.6, MICU 4.7, CCU 4.9; *p *= 0.27) is meaningful. This suggests that even though the patients were located in different ICUs (thus presuming different diagnoses); their response to the same IIP was effective. Interestingly, the only difference in patient blood glucose > 150 mg/dL was found at 24 hours between STICU (13%), MICU (17%), and CCU (36%); *p *< 0.001. Although statistically significant, this may not be clinically significant given that approximately half of the CCU patients were not on the IIP at 24 hours. Between patient variability is indicated by the width of the box plots show in Figures 4, 5, 6. For MICU (Figure 5) and CCU (Figure 6) patients, time 0 blood glucose is highly variable but decreases over time. STICU (Figure 4) patients are not as variable at time 0 and have less decrease over time.

Furthermore, mean insulin drip rates, time to goal, and deviation from IIP all were clinically similar and not statistically significant which may suggest that patient differences, not protocol adherence, may be the contributing factor that may account for our findings. Although we did not look for absolute deviations, we cannot rule these out. We also acknowledge the potential that investigators could have biased the outcome related to deviations due to presence on the unit. It appears that in critically ill patients, glucose levels rather than amount of insulin infused account for mortality benefit [25]. The Van den Berghe study [1] found reductions in mortality and morbidities attributable to mean glucose of 103 mg/dl in the intensive treatment group compared to the more modestly treated group with mean glucose value of 153 mg/dL. Furthermore, the findings appeared most dramatic in the patients who had ICU stays of greater than 5 days. Limitations of Van den Berghe's study include questionable generalizability of results outside of the cardiac surgical population, single center study with unclear feasibility in a larger scope, and early aggressive glucose administration for all patients in both treatment and control groups which may have forced some of the hyperglycemia and insulin dosing. However, the study has largely been embraced as evidence for intensive insulin therapy in the ICU.

Our patient characteristics do show a difference between age, diabetes, steroid, and beta blocker use which may contribute to each ICUs difference in blood glucose value at initiation of IIP. Moreover, ICU length of stay and IIP duration was greater in the STICU compared to MICU and CCU. Because of this time difference, comparing mean blood glucose values was not pertinent because of the correlation to length on the IIP (the longer on the IIP, the great chance to get into goal range). Hyperglycemia was a risk factor in non diabetic patients admitted in cardiac, cardiothoracic and neurosurgical ICUs, however not in medical or general surgical ICUs [7]. In addition, intraoperative hyperglycemia was found to be an independent risk factor for complications following cardiac surgery [12]. Although a recent study [14] revealed insulin therapy has been shown to reduce morbidity in medical ICU patients. This lends the importance of examining different patient populations who may be at risk for hyperglycemia.

Most nurse initiated protocols identified thus far in the literature have a component where the RN needs to perform some method of calculation to derive the insulin rate to be infused [16-18,21,22]. The use of our non-calculated IIP in this study, contributes to the safety and standardization of the protocol. It appears our 4-column protocol adapts to glucose sensitive and resistant patients in a similar way in multiple patient populations. In addition, use of a nurse initiated IIP has significant impact on nursing practice by demonstrating decision making and critical thinking skills by the RN in the care of the critically ill patient.

The concern regarding the risk of hypoglycemia with intensive insulin protocols has limited the expansion to general care areas. However, the incidence of severe hypoglycemia ( < 40 mg/dL) of patients on a protocol was similar in both critical care (4%) and non-critical care (3.6%) areas [20]. In addition, hypoglycemia ( < 45 mg/dL) was not associated with mortality, although noted to be a small study [26]. A systematic review of twenty-four reports suggest that tight glucose control protocols maintained adequate glycemic control and resulted in low frequencies of hypoglycemia [23]. Our study also had a more conservative definition of hypoglycemia ( < 60 mg/dL) with similar incidence across the 3 ICUs. Survivors within the 48 hours after IIP discontinuation or patient transfer, patients had significant increase in mean percent of blood glucose values > 150 mg/dL (*p *< 0.0001). Given limited hypoglycemia risks and our results related to hyperglycemia after protocol discontinuation or patient transfer, it would seem prudent to research the use of IIP in general care areas and explore this transition further.

The investigators acknowledge several limitations with this study. It was conducted in one setting with a lack of randomized, controlled design. There were patient differences between and within population groups that were not controlled for and a convenience sample of patients who met inclusion criteria was utilized. We acknowledge variability with utilizing three different types of blood glucose measurements. At the time of the study, common practice was to perform measurement with a capillary sample. Extreme values (BG > 400 mg/dL and < 60 mg/dL) were verified with an additional venous or arterial sample. We have subsequently changed to utilizing arterial samples, when arterial line is in place. We only use capillary blood glucose in occasions of no arterial line, based on ours and others analysis of variances in the extremes of glucose levels associated with capillary analysis [27]. In addition, there were different RNs implementing and following the protocol and the demographic and transfer data was collected by different data collectors. The lack of fidelity or integrity measures to assure nurses were implementing the interventions accurately and reliably is also recognized. Based on findings from this study related to IIP safety, current research literature, and input from physicians, pharmacists and nurses at our institution, a revised 3-column IIP with a tighter goal range for blood glucose levels of 80–130 mg/dL has been implemented and is available for use in any adult ICU. Although it is recognized that oversimplification of the initial 4-column approach may lead to differentiation of outcomes in different populations.

## Conclusion

The RN initiated IIP was effective and safe in achieving a target glucose goal of 100–150 mg/dL in the surgical, cardiac and medical ICUs in our institution. The key to successful IIP use involved multidisciplinary team approach of nurses, pharmacists, physicians and laboratory personnel. No further tailoring of this protocol is needed for use amongst differing patient groups. Control of blood glucose post IIP had marked variability suggesting the need for further studies regarding protocolized glucose control of patients transitioning out of the ICU.

## Competing interests

The author(s) declare that they have no competing interests.

## Authors' contributions

MB conceived of the study, participated in its design, carried out the education of the protocol, participated in the data collection, entered some data for analysis, coordinated and drafted the manuscript. LO conceived of the study, participated in its design, and helped draft the manuscript. KW, JE, LE, AT, conceived of the study, participated in study design, carried out the education of the protocol, participated in the data collection and helped draft the manuscript. PK, MB, OG, JCF participated in study design and helped draft the manuscript. All authors read and approved the final manuscript.

## Pre-publication history

The pre-publication history for this paper can be accessed here:



## Supplementary Material

Additional file 1 Insulin Infusion Protocol. The image provided represents the insulin infusion protocol utilized for the study. By permission of Mayo Foundation for Medical Education and Research. All rights reserved. Click here for file
