# Supplementary figures and images for: Comparison of a nurse initiated insulin infusion protocol for intensive insulin therapy between adult surgical trauma, medical and coronary care intensive care patients
Source: BMC Emerg Med. 2007 Aug 29;7:14. doi: 10.1186/1471-227X-7-14 (PMC2064915; doi:10.1186/1471-227X-7-14)

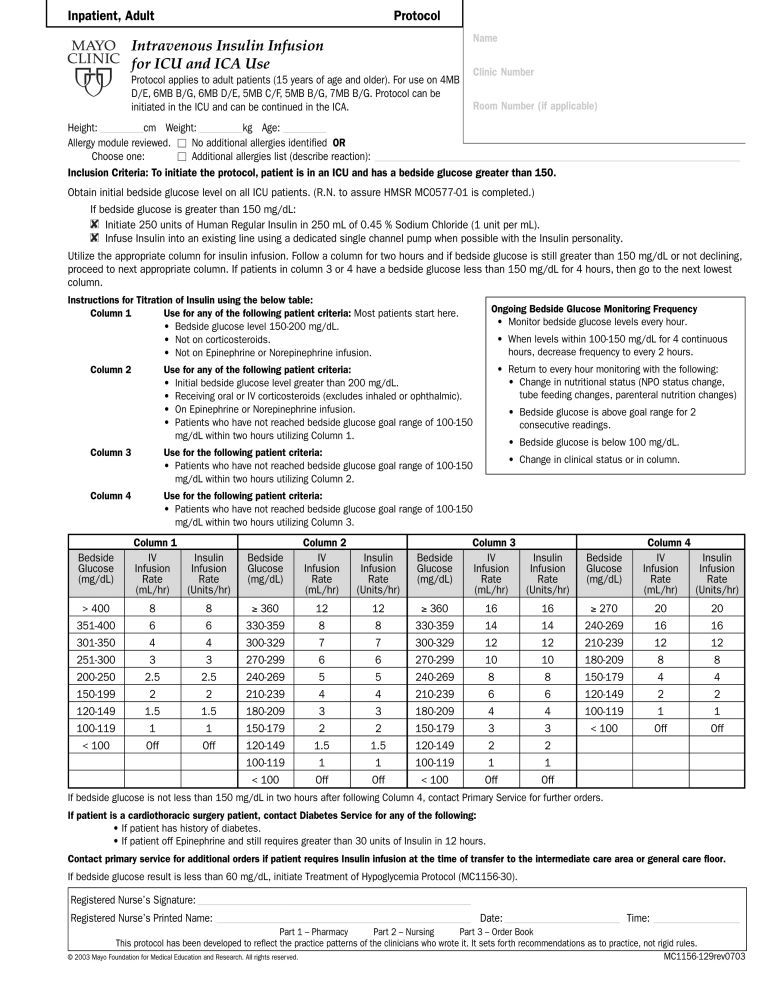

Supplement: Additional file 1 — Insulin Infusion Protocol. The image provided represents the insulin infusion protocol utilized for the study. By permission of Mayo Foundation for Medical Education and Research. All rights reserved. [file 1471-227X-7-14-S1.jpeg]
